# Physiological electric field works via the VEGF receptor to stimulate neovessel formation of vascular endothelial cells in a 3D environment

**DOI:** 10.1242/bio.035204

**Published:** 2018-09-15

**Authors:** Yihong Chen, Liyan Ye, Linbo Guan, Ping Fan, Rui Liu, Hao Liu, Jinxin Chen, Yue Zhu, Xing Wei, Yu Liu, Huai Bai

**Affiliations:** 1 Laboratory of Genetic Disease and Perinatal Medicine and Key Laboratory of Birth Defects and Related Diseases of Women and Children of the Ministry of Education, West China Second University Hospital, Sichuan University, Chengdu 610041, Sichuan, P.R. China; 2 Department of Obstetrics and Gynecology, West China Second University Hospital, Sichuan University, Chengdu 610041, Sichuan, P.R. China; 3 Division of peptides related with human disease, West China Hospital, Sichuan University, Chengdu 610041, Sichuan, PR China; 4 Department of Biochemistry and Molecular Biology, West China School of Preclinical and Forensic Medicine, Sichuan University, Chengdu 610041, Sichuan, P.R. China

**Keywords:** Angiogenesis, Endothelial cells, Electrical stimulation, 3D, Tube formation

## Abstract

Electrical stimulation induces significant neovessel formation *in vivo*. We have shown that electrical stimulation of endothelial cells functions as an important contributor to angiogenesis in monolayer culture. Because angiogenesis occurs in a three-dimensional (3D) environment, in this study we investigated the effects of a direct current (DC) electrical field (EF) on endothelial neovessel formation in 3D culture. There was a significant increase in tube formation when endothelial cells were stimulated with EF for 4 h. The lengths of the tube-like structures were augmented further by the continued EF exposure. The lengths of the tubes also increased dose-dependently in the EF-treated cultures in the field strengths of 50 mV/mm∼200 mV/mm for 6 h. Electrical fields of small physiological magnitude enhanced VEGF expression by endothelial cells in 3D culture. EF treatment also resulted in activation of VEGFR2, Akt, extracellular regulated kinase 1,2 (Erk1/2), as well as the c-Jun NH2-terminal kinase (JNK). The tyrosine kinase inhibitor SU1498 that blocks VEGFR2 activity exhibited a potent inhibition of tube growth, and the Akt inhibitor MK-2206 2HCl, the Erk1/2 inhibitor U0126 and the JNK inhibitor SB203580 significantly reduced EF-stimulated tubulogenesis. These results suggest the importance of the VEGFR2 signaling pathway during EF-induced angiogenesis. The results of this study provide novel evidence that endogenous EFs may promote blood vessel formation of endothelial cells by activating the VEGF receptor signaling pathway.

## Abstract (precis)

**Summary:** This study reports the effects of a direct current electrical field on endothelial neovessel formation in 3D culture. Endogenous EFs may promote angiogenesis of endothelial cells by activating the VEGF receptor signaling pathway.

## INTRODUCTION

Blood vessel formation (angiogenesis) and tissue vascularization play a vital role in many important physiological and pathological processes, including embryogenesis, wound healing and the growth of solid tumors (Conway et al., 2001; Carmeliet, 2005; Carmeliet and Jain, 2000). Vascular endothelial cells (ECs) are a fundamental cell type involved in such processes. EC cell migration, elongation and alignment are early events in angiogenesis. Later, the cells organize into a tubular network and form new blood vessels. Such cellular processes are influenced by the micro-environment in which the cells reside. *In vivo*, both biochemical and physiological cues are involved in regulating cellular functions (Conway et al., 2001; Ingber, 2002; Hudlicka, 1998; Song et al., 2002). Targeting angiogenesis is becoming widely accepted as a potential therapy for various diseases. For example, promoting neovessel formation in ischemic hearts could benefit patients with coronary heart disease, whereas inhibiting tumor neovessel formation could inhibit tumor growth and development.

Physiological electric fields (EFs) occur in embryonic development and wound healing (Robinson, 1985; Robinson and Messerli, 1996; McCaig and Zhao, 1997) in which active angiogenesis is taking place. Several studies have suggested that EFs regulate neovessel formation. For instance, EFs were shown to stimulate contraction of skeletal muscles to induce neovessel formation and VEGF production (Kanno et al., 1999), and an under-threshold EF also showed a similar effect in rat skeletal muscle (Nagasaka et al., 2006). *In vitro* studies have shown that EFs not only reorient endothelial cells but also stimulate cell migration and elongation. These three cell behaviors are all forerunners of angiogenesis (Zhao et al., 2004; Bai et al., 2004). In addition, EFs also stimulate growth factor secretion and/or expression that guide the cell behaviors (Zhao et al., 2004; Bai et al., 2011). Until now, the *in vitro* effects of a DC EF on endothelial cells have been based on two-dimensional (2D) culture models (Zhao et al., 2004; Bai et al., 2004, 2011) or on gel culture models (Tzoneva et al., 2016). Although these data suggest a role for EFs in essential aspects of neovessel formation, these *in vitro* studies were not carried out in the context of angiogenesis, where diverse cellular activities stimulated by EFs proceed through a highly coordinated spatiotemporal sequence of events in a 3-dimensional (3D) environment. Besides, several other forms of EFs, such as amplitude EF and pulsed electromagnetic fields, on endothelial cells, either using gelatin-based on-top culture (Sheikh et al., 2013) or microcarrier fibrin gel culture, have been reported (Tepper et al., 2004).

While these findings suggest that EFs induce neovessel formation, further investigation is required because EFs represent a new and promising paradigm for controlling angiogenesis, and mechanistic insights can be obtained through *in vitro* study. To date, the exact EF-induced angiogenic effect and its underlying molecular mechanisms remain unclear.

In this study, we used *in vitro* 3D culture to directly examine the effect of DC EF on the capacity of endothelial cells to form tubular networks over a period of time. The study found that the organization of tube-like structures is affected by EF stimulation, and the VEGF/VEGF receptor (VEGFR) signaling pathway is responsible for the EF-induced effect. This study provides an understanding of the role of EF in the regulation of angiogenesis.

## RESULTS

### Effect of EFs on tube formation

**Fig. 1.**
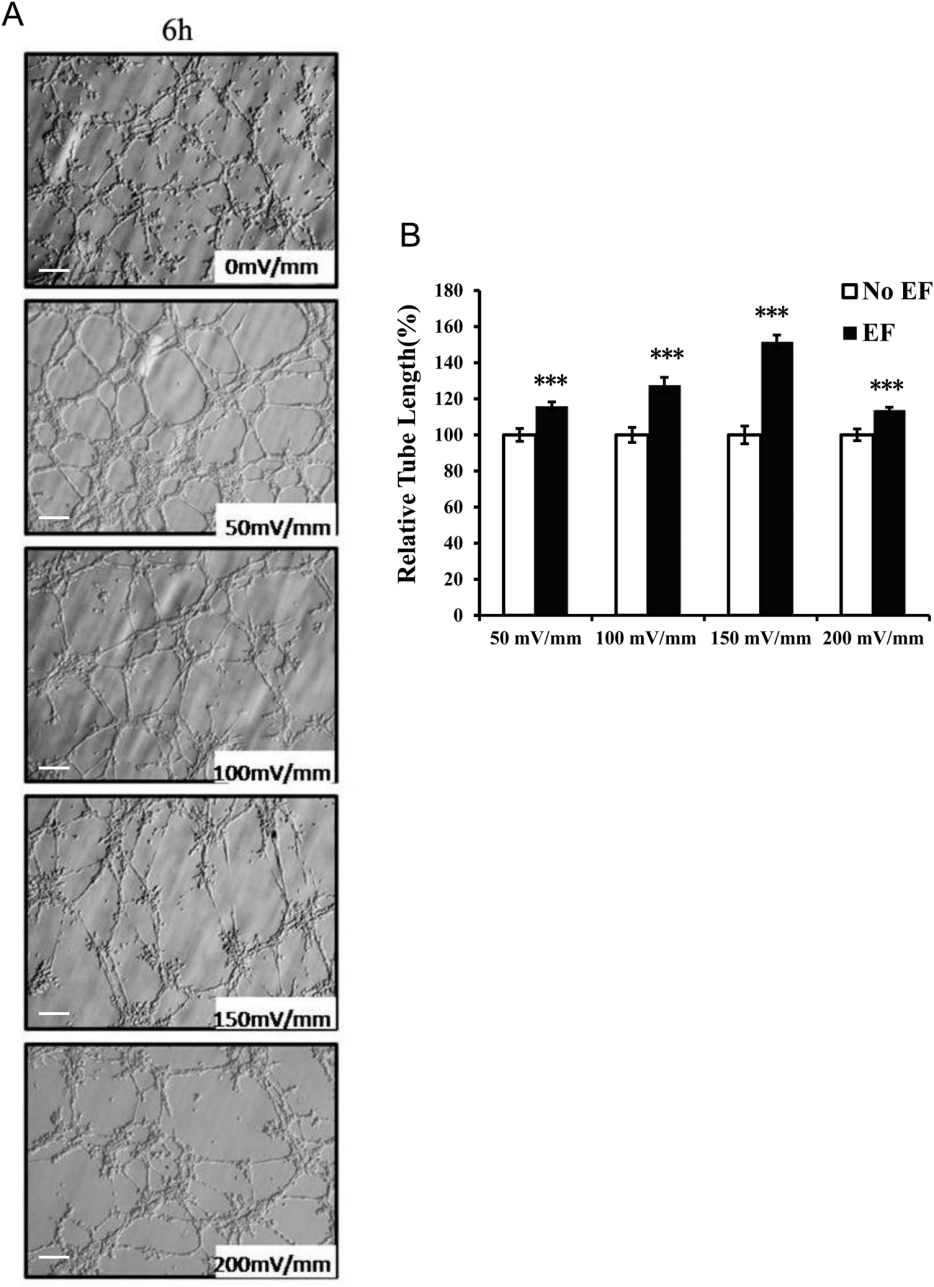
**Proangiogenic activities of endothelial cells response to different EF strengths.** Voltage dependence of EF-enhanced tube formation of endothelial cells (A). The relative tube length of HUVECs cultured in 3D (see the Materials and Methods) was calculated during a 6 h period. The tube length enhancement of HUVECs was voltage dependent (B). The error bars represent the S.E. ***P<0.001, when compared with the no EF control (0 mV). Initial magnification of the images: 200X. Scale bar: 100 μm.

**Fig. 2.**
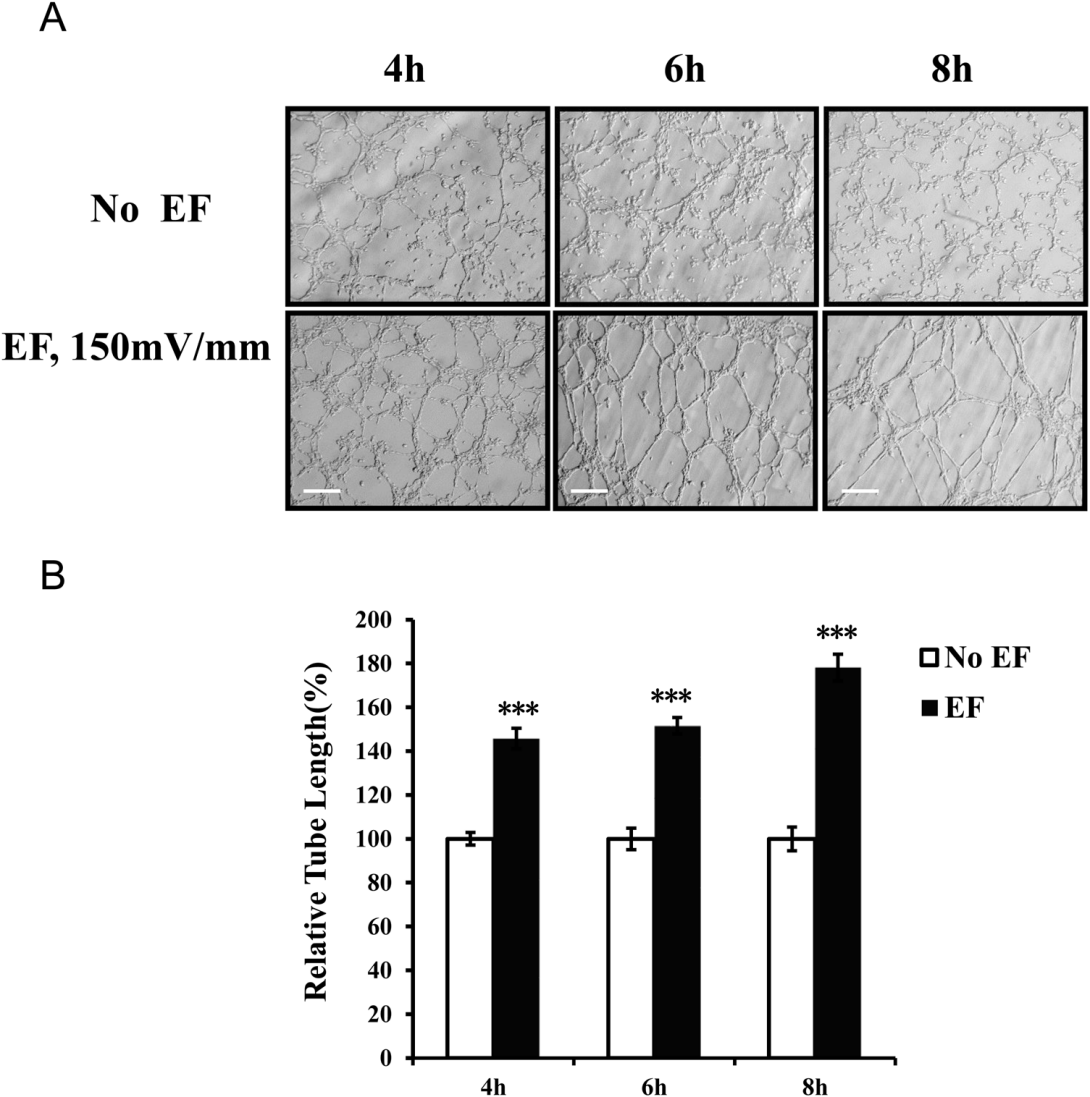
**Effect of EF treatment at different time points on proangiogenic activities of endothelial cells.** Time dependence of EF-enhanced tube formation of endothelial cells (A). The relative tube length of HUVECs cultured in 3D (see the Materials and Methods) was calculated during a 4–8 h period. The tube length enhancement of HUVECs was time dependent (B). The error bars represent the S.E. ***P<0.001, when compared with the no EF control (0 mV). Initial magnification of the images: 200X. Scale bar: 100 μm.

In this study, we used a 3D Matrigel model to evaluate the EF-stimulated tube formation of HUVECs. The maximal tube formation as measured by tube length was observed in experiments utilizing an EF of 150 mV/mm stimulation (Fig. 1). The total length of the tubular network was significantly increased in the presence of EF exposure (150 mV/mm) compared to the length in the control (*P*<0.001). Tube formations at field strengths of 50, 100 or 200 mV/mm were also significantly enhanced as compared to those of the corresponding controls (all *P*<0.001) (Fig. 1). The EF-induced tubulogenesis was also time dependent and increased in the 4 h to 8 h period (Fig. 2).

### Increase in VEGF expression following electrical field treatment of endothelial cells in 3D culture

**Fig. 3.**
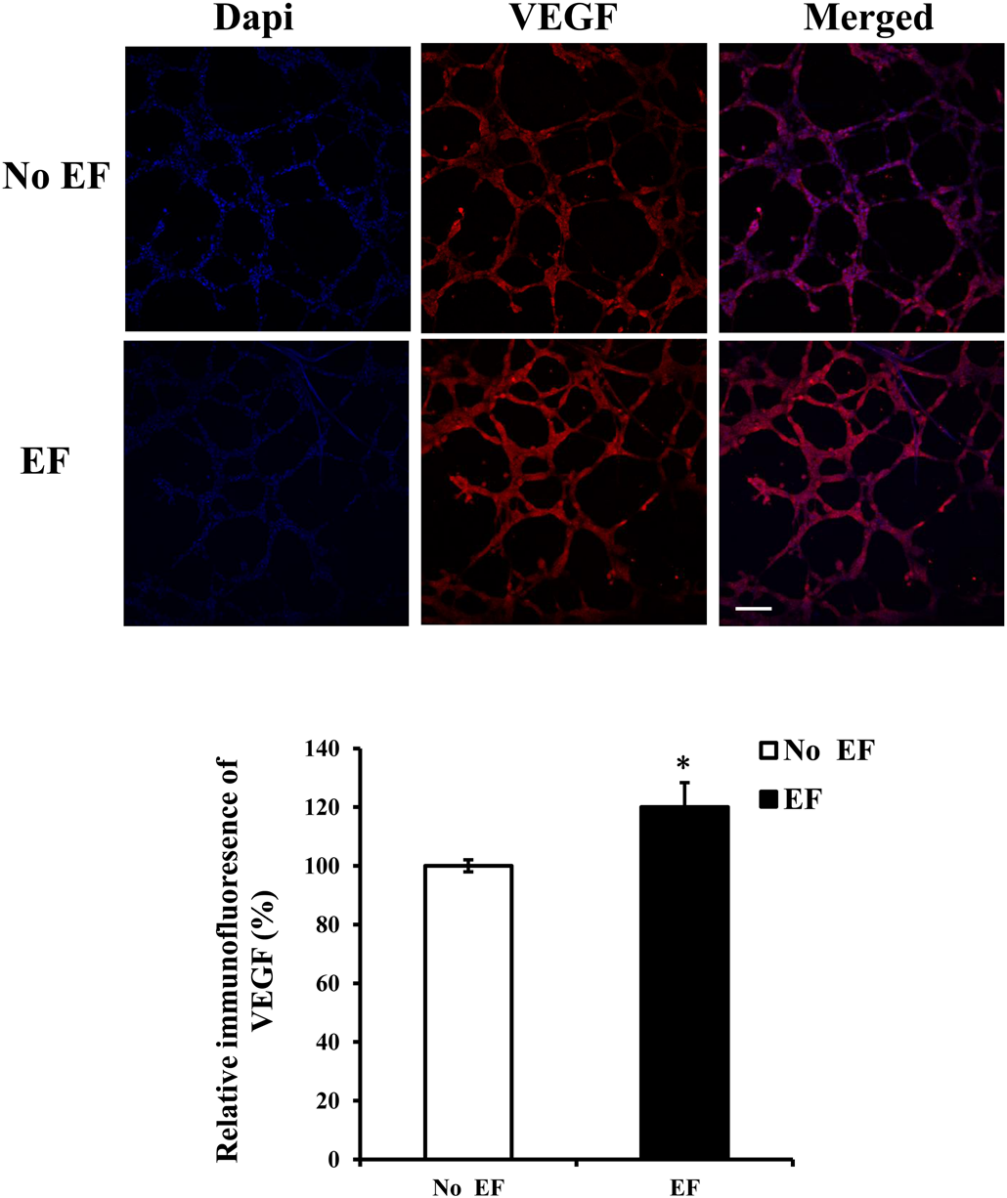
**Increase of VEGF expression upon EF treatment of HUVECs cultured in 3D.** HUVECs were treated with an electrical field (150 mV/mm). After 6 h, they were fixed and stained with a VEGF antibody. Protein expression was quantified by confocal laser scanning microscopy. The images show representative immunolabeled tube-like structures. The histogram depicts the relative immunofluorescence of the VEGF protein. The error bars represent the S.E. *P<0.05, significantly different from the untreated control. Initial magnification of the images: 200X. Scale bar: 100 μm.

Since the EF-induced angiogenic response of endothelial cells in a 2D environment requires increased expression of VEGF (Zhao et al., 2004), an increase in VEGF expression in 3D culture should be assumed. To examine this hypothesis, endothelial cells were stimulated with a field strength of 150 mV/mm for 6 h, and VEGF protein expression was detected. Supporting our previous observation (see Figs 1 and 2), there was a significant elevation of VEGF (120.4% of the untreated control level, which was set to 100%) in the sample for 6 h of electrical field exposure (Fig. 3) (*P*<0.05).

### Effects of EFs on VEGFR2 phosphorylation and activation of downstream signaling pathways

**Fig. 4.**
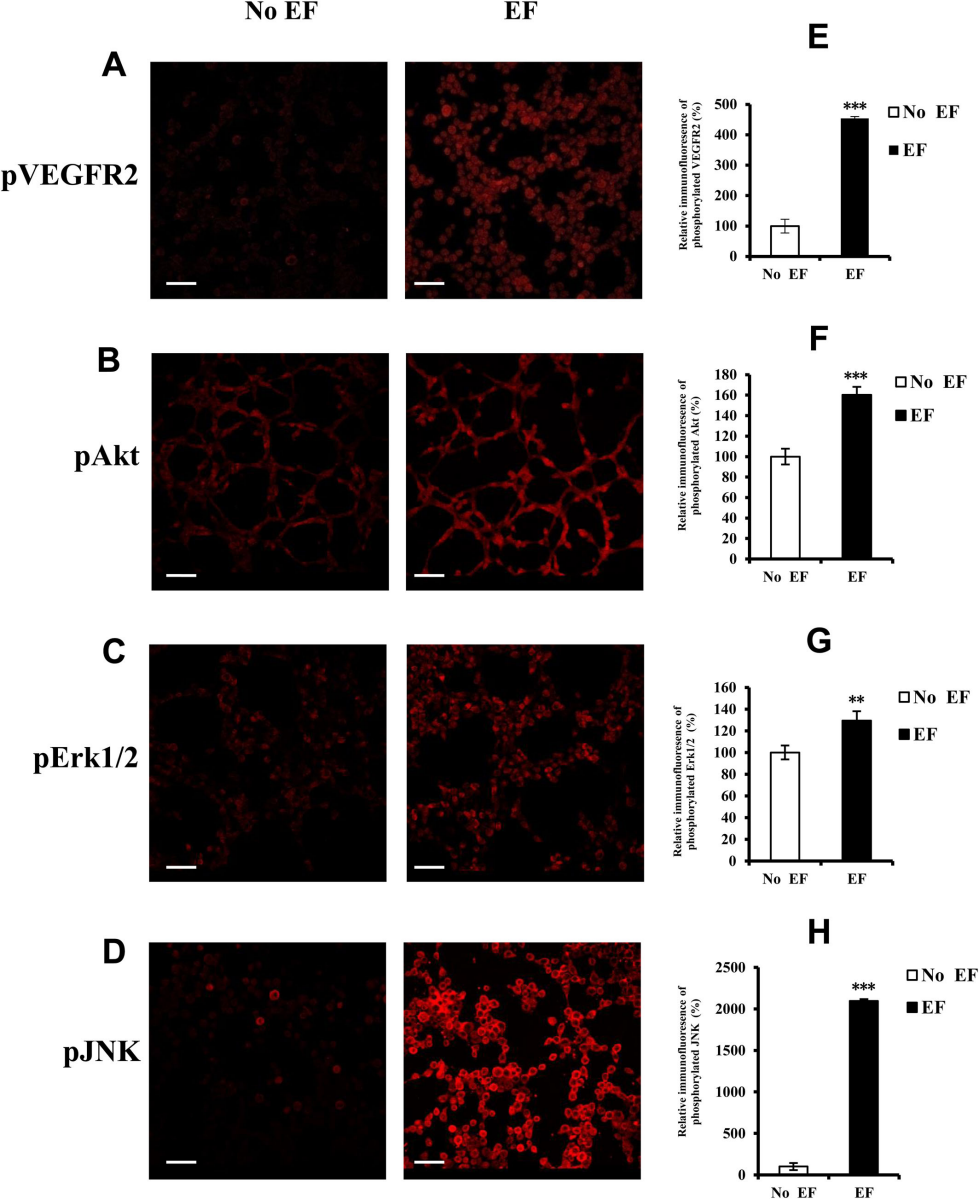
**Activation of VEGFR2, Akt, Erk1/2 and JNK following EF treatment.** Tube-like structures of endothelial cells cultured in 3D were treated with an EF (150 mV/mm). After 15 min, they were fixed and stained with antibodies directed against the active (phosphorylated) form of the proteins. Protein expression was quantified by confocal laser scanning microscopy. The images show representative immunolabeled tube-like structures (A–D). The histogram depicts the relative immunofluorescence of the phosphorylated proteins (E–H). The error bars represent the S.E. **P<0.01, ***P<0.001, significantly different from the untreated control. Initial magnification of the images: 200X. Scale bar: 100 μm.

The stimulation of angiogenesis may be responsible for the electrical field stimulated activation of the VEGFR2 and its down-stream signaling pathway which have previously been shown to be involved in the EF-induced preangiogenic (early events of neovessel formation) response of endothelial cells in 2D culture (Zhao et al., 2004). Therefore, phospho-specific antibodies were used to measure the activation of VEGFR2, Akt, and the MAPKs Erk1/2, p38 and JNK after electrical field treatment. Electrical field treatment led to the activation of VEGFR2, Akt, Erk1/2, and JNK at 15 min (Fig. 4).

### Effects of VEGFR2, Akt and MAPK inhibitors on EF-induced vessel-like structure formation

**Fig. 5.**
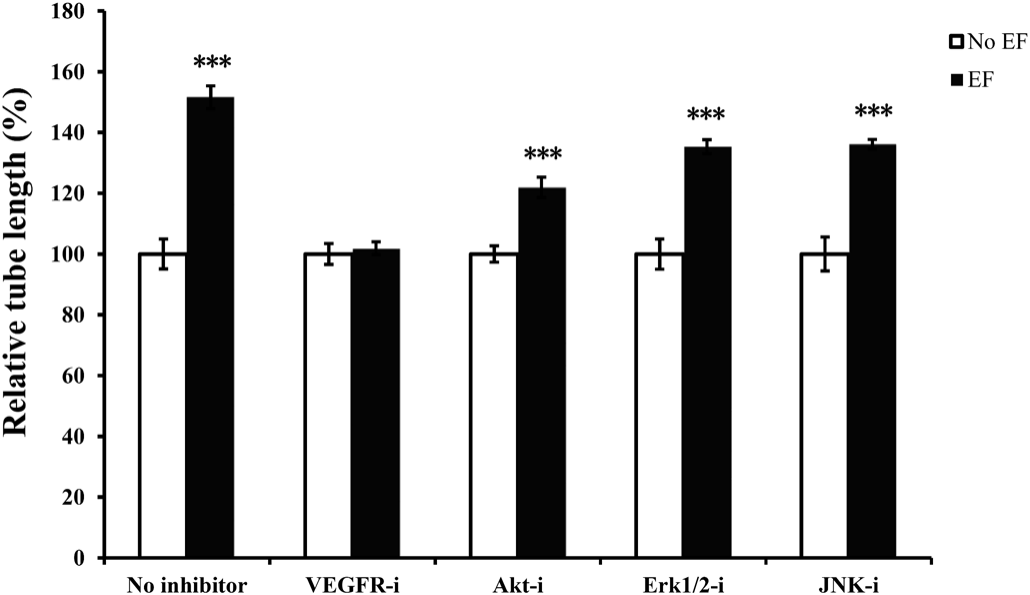
**Effects of various drugs on EF-induced tube formation of endothelial cells.** Inhibition of Akt (Akt-i), Erk1/2 (Erk1/2 -i) and JNK (JNK-i) significantly decreased tube length, whereas inhibition of VEGFR2 (VEGFR2-i) potently abolished the EF-mediated enhancement of tube length. The tube lengths were expressed as a percentage relative to that obtained in the untreated control in EF culture. VEGFR-i, VEGFR inhibitor SU1498 (50 μM); Akt-i, Akt inhibitor MK-2206 2HCl (10 μM); Erk 1/2-i, Erk 1/2 inhibitor U0126 (20 μM); JNK-i, JNK inhibitor Sp600125 (10 μM). Endothelial cells cultured in 3D were subjected to EFs of 150 mV/mm for 6 h. Each treatment was performed in duplicate in at least three independent experiments. The error bars represent the S.E. ***, *P*<0.001 compared to cells exposed to 150 mV/ mm without drug treatment.

To verify that the formation of vessel-like structures requires VEGF receptor activation, endothelial cells were incubated with the specific VEGFR2 antagonist SU1498 (50 µM). This significantly reduced the EF-induced increase in tube length and has the inhibition rate of 56.0% (Fig. 5).

To investigate the specific role of Akt and MAPKs on tube formation, endothelial cells in 3D culture were treated in the absence or presence of an EF with either the Akt inhibitor MK-2206 2HCl (10 μM), Erk1/2 inhibitor U0126 (20 μM), or JNK inhibitor Sp600125 (10 μM). These treatments also significantly inhibited EF-induced tubulogenesis with the inhibition rate by 44.9%, 39.7% and 36.4%, respectively (Fig. 5, all *P*<0.001). None of the inhibitors displayed toxic effects or retarded growth in cell culture (data not shown).

## DISCUSSION

It has been documented that DC electrical fields provide biophysical cues that modulate the early events of angiogenesis in endothelial cells (Zhao et al., 2004; Bai et al., 2004). In the present study, we provided the first evidence that DC EFs promote vascular tube formation from endothelial cells in 3D culture conditions, suggesting that EFs not only induce the early events of angiogenesis from ECs but also have an integrative role during sprouting tubular formation. In addition, we also found that the VEGF-mediated activation of VEGFR2 and its downstream pathways is involved in the EF-enhanced tubulogenic process.

*In vitro* functional studies are commonly done using cells growing in a 2D monolayer. However, 3D culture enables cells to grow in a more biologically relevant environment that better simulates *in vivo* conditions (Pampaloni et al., 2007). At present, synthetic scaffolds or gels of biological or synthetic origin (Tibbitt and Anseth, 2009) are typically used to create the 3D culture model. Matrigel, a basement membrane extract from a mouse sarcoma, is the most commonly used biological scaffold and is enriched in collagen IV, laminin, and various growth factors (Kleinman and Martin, 2005). Cell growth with these scaffolds more closely resembles growth *in vivo*, while allowing for better phenotypic and functional observations of the cells.

Quantitative analysis of total tube length demonstrated that there was a significant increase in tube formation when HUVECs were stimulated with EF for 4 h (Fig. 2). The lengths of the tube-like structures were augmented further by the continued EF exposure (Fig. 2). EF exposure did not have detrimental effects on the tubular network structures within the observed period of time. The lengths of the tubes also increased in the EF-treated cultures in the field strengths of 50 mV/mm∼200 mV/mm for 6 h (Fig. 1). EF strength at 150 mV/mm gave rise to the highest growth rate. These results suggest that EFs have obvious time and voltage dependent effects. The relative increase rate of tubes at higher voltage (200 mV/mm) is lower than that of lower voltage (50, 100 and 150 mV/mm) might be attributed to the EF-induced inhibiting effect, since the EF experimental system, as we have used, has been proved to be stable in studying cellular responses to DC electric signals (Song et al., 2007), and there has been no noticeable detrimental effect of tube-like structure in the culture. Further study will help us understand the mechanisms underlying the inhibitory effect.

VEGF is one of the most potent angiogenic factors and promotes many of the events necessary for angiogenesis, including the proliferation and migration of vascular endothelial cells, remodeling of the extracellular matrix and the formation of capillary tubules both *in vitro* and *in vivo* (Ferrara, 1995; Matsunaga et al., 2008). Neutralizing antibodies against VEGF and expression of antisense VEGF blocked neovessel formation (Kim et al., 1993; Kendall and Thomas, 1993; Saleh et al., 1996). VEGF is a potent pro-angiogenic factor with a key role in several steps of angiogenesis.

A previous study has shown that EF enhances VEGF secretion in a monolayer culture model. Electrical stimulation also increased secretion and expression of VEGF from endothelial cells, which did not require any other type of cells (Zhao et al., 2004; Bai et al., 2011). HUVECs grown in the presence of DC EFs (150 mV/mm) produced significantly more VEGF than unstimulated control cultures (Zhao et al., 2004). Pulsed electrical stimulation also induced significant VEGF expression in embryoid bodies derived from mouse embryonic stem cells (Sauer et al., 2005). Interestingly, DC EF or high-frequency EF induced an increase in VEGF production by HUVECs or microvascular endothelial cells on gelatin-based hydrogels (Tzoneva et al., 2016; Sheikh et al., 2013), whereas pulsed electromagnetic fields (PEMF) stimulated another proangiogenic factor FGF-2 production but not VEGF secretion by HUVEC in microcarrier fibrin gel culture (Tepper et al., 2004). In this study, we extended these findings and demonstrated that DC EFs have a role in stimulating VEGF expression in a 3D culture model of angiogenesis.

VEGF binding to VEGF receptors triggers a core signal transduction cascade that promotes neovessel formation (Carmeliet and Jain, 2000; Ferrara, 1995; Risau, 1997; Folkman, 2007) by stimulating the above described angiogenic cell behaviors (events). Studies demonstrated that the VEGFR and its downstream components were involved in EF-induced angiogenic phenotypes using an EC-monolayer cultures (Zhao et al., 2004) or an embryonic stem cell model (Sauer et al., 2005). In this study, DC EFs activated VEGFR2, and VEGFR2 inhibitors potently blocked the EF-induced formation of endothelial tubes in a 3D culture model. These results support prior studies from our group and from others (Zhao et al., 2004; Bai et al., 2011; Sauer et al., 2005).

How EFs induce VEGF expression is not clear. Because the reactive oxygen species (ROS) scavenger, vitamin E, significantly decreased electrically-induced VEGF expression, one study suggested that ROS may mediate the VEGF increase (Kendall and Thomas, 1993). We have investigated the changes in the protein secretion and transcription of VEGF and IL-8 that were induced by DC electrical stimulation in an EC-monolayer model. The levels of VEGF protein and mRNA were significantly increased following electrical stimulation (Zhao et al., 2004; Bai et al., 2011). The present study provides further evidence that EFs enhance the expression of the proangiogenic factor VEGF in a 3D environment. Of note, in rabbit skeletal muscle, the frequency of EF stimulation had a significant influence on the production of VEGF and HIF-1 alpha proteins (Shen et al., 2009).

The results of our study showed that EFs stimulated VEGF receptor activation in 3D culture. Activation of VEGF receptor leads to the activation of its downstream signaling pathways, including PI3k/Akt kinase, MAP kinases (Erk1/2, JNK), PKC, and calcium in vascular endothelial cells (Cross et al., 2003; Thakker et al., 1999). This study demonstrated significant activation of some VEGF receptor downstream components, including Akt, Erk1/2 and JNK, indicating EF-induced signal transduction. This was further confirmed by experiments in which VEGFR2, Akt, Erk1/2 and JNK inhibitors significantly caused inhibition of the EF-induced tubulogenic effect of endothelial cells in 3D culture conditions. None of these inhibitors were able to entirely block tubulogenesis. These results suggest that the VEGFR2 is involved and Akt-, Erk1/2- and JNK-activated states are also implicated in the EF-induced tubulogenesis.

While we and others found that VEGF receptor signaling is associated with angiogenic potential or tube formation of ECs, which represents an important mechanism in EF-induced angiogenesis, our preliminary study shows that other signaling molecules such as bFGF, IGF and PDGF might also be involved in the endothelial tube formation under DC EF stimulation (Fig. S1), which is in line with the report that pulsed electromagnetic fields augments angiogenesis by stimulating endothelial release of FGF-2(bFGF) (Tepper et al., 2004). It warrants further study in order to determine down-stream cascades of these molecules in the EF-induced effect and the role of these signaling pathways in EF-induced tube formation, and determine interaction (or crosstalk) of VEGF/VEGFR2 and these pathways involved in electric stimulated angiogenic enhancing potential of endothelial cells.

It is well documented that DC EFs provide unique directional cues for cellular behaviors, including the directional migration of ECs in culture (Zhao et al., 2004; Bai et al., 2011). Such a feature is difficult to study *in vivo*. In a preliminary study, we cultured mouse aortic rings in an electrotaxis chamber (Fig. S2) and applied EFs that induced directional formation and growth of vessel-like structures towards the anode (Fig. S3). These data suggest that EFs can play a critical role in enhancing and directing the growth of angiogenic tubular structures.

It is interesting to note that there are several reports showing EF induced endothelial responses, which differed from the present study using DC EF in 3D culture condition. For instance, a study using cells on-top culture of gelatin-based hydrogels showed enhanced cell attachment, VEGF production, fibronectin (FN) synthesis and MMP-2 and -9 activity of HUVEC and MDA-MB-231 cells under DC EF culture (200 mV/mm) (Tzoneva et al., 2016). This study did not show the effect of EF-induced angiogenic morphology. Another study reported that high-frequency EF enhanced capillary morphogenesis, VEGF release, MEK and Erk phosphorylation using on-top peptide nanofiber hydrogel culture model (Sheikh et al., 2013). Tepper and colleagues, using *in vitro* microcarrier fibrin gel model and *in vivo* matrigel plug assay, revealed that pulsed electromagnetic fields (PMEF) increased endothelial cell tubulization, proliferation, FGF-2 production and angiogenesis (Tepper et al., 2004). These studies, together with our current findings, suggest that EF induced angiogenesis might be of true biological relevance *in vivo*.

Modern electrical stimulation therapies have shown to be effective for chronic pain management and may promote the healing of bone fractures and chronic wounds (Fleischli and Laughlin, 1997). Interestingly, two recent reports showed that long term functional electrical stimulation produced positive modulation of electrostimulated epidermis, that correlates with significant improvements in muscle size and function in spinal cord injury patients with denervated muscles (Albertin et al., 2018a,b). Based on the present study and reports from others (Patterson and Runge, 1999), it can be speculated that electrical stimulation may be another effective method for accelerating angiogenesis, and studies to evaluate its effectiveness in humans with ischemic vascular disease will potentially open the door in the search for novel approaches to treat ischemic diseases.

In conclusion, this study provided the evidence of physiological level of DC EFs stimulating the neovessel formation of endothelial cells in 3D culture, which extends our previous findings from a monolayer model. We suggest that one of the main mechanisms through which the EF confers its effect is VEGF activation of VEGFR-2-mediated signaling pathways that control endothelial migration and proliferation. Our findings encourage the design of *in vivo* study to explore the effects of electrical fields on enhancing and guiding angiogenesis.

## MATERIALS AND METHODS

### Cell cultures and reagents

The human umbilical vein endothelial cell (HUVEC) line from ATCC was used (Bai et al., 2011). The HUVECs were maintained in Dulbecco's modified Eagle's medium (DMEM) supplemented with 10% fetal bovine serum (FBS), 2 mM L-glutamine, penicillin (50 units/ml), and streptomycin (50 µg/ml) at 37°C in 5% CO_2_ (Zhao et al., 2004). Matrigel was from BD Biosciences. Primary antibodies against Akt, Erk1/2 and p38 MAPK (active form) were purchased from Cell Signaling Technology, and VEGF, VEGFR2 and JNK (active form) antibodies were purchased from Abcam. The DyeLight 680-labeled secondary antibody to rabbit IgG (H+L) was the product of KPL (Milford, USA). The VEGFR inhibitor SU1498 (for VEGFR2 or KDR), Akt inhibitor MK-2206 2HCl, Erk1/2 inhibitor U0126 and JNK inhibitor Sp600125 were from Abcam or Selleckchem (Houston, USA).

### 3D cultures and electrical stimulation

3D cultures were prepared by implanting cells in Matrigel using a two-step procedure. First, cells were cultured on growth factor-reduced Matrigel (BD Biosciences) using the overlay method (Debnath et al., 2003), and then a thin layer of the Matrigel (50 µl) was applied. After an incubation period of 2 h at 37°C in 5% CO2, the cells were exposed to an EF. The EF exposure protocols were similar to those reported previously (Zhao et al., 1996) (Fig. S2) with minor modification. In brief, for tube-like structure analysis or for protein expression or activation analysis, 10 (Song et al., 2002) vascular endothelial cells/ml were seeded in Matrigel as described above in a specially made trough formed by two parallel (2 cm apart) strips of glass coverslips (No. 1, length of 22 mm or 50 mm) fixed to the base of the dish with silicone grease (Dow Corning, DC4, Midland, USA). A No. 1 coverslip roof was applied and sealed with silicone grease. The final dimensions of the chamber, through which current was passed, were 22×10×0.4 mm. Agar-salt bridges not less than 15 cm long were used to connect silver/silver-chloride electrodes in beakers of Steinberg's solution [58 mM NaCl, 0.67 mM KCl, 0.44 mM Ca(NO3)2, 1.3 mM MgSO4, 4.6 mM Trizma base, pH 7.8–8.0], to pools of excess culture medium at either side of the chamber. This prevents diffusion of electrode products into the culture medium. EF strengths in the physiological range of 50, 100, 150 and 200 mV/mm were used. Field strengths were measured directly at the beginning, the end, and during each experiment. No fluctuations in field strength were observed. For drug inhibition experiments, the cells were incubated with the VEGFR2 inhibitor SU1498 (50 μM), Akt inhibitor MK-2206 2HCl (10 μM), Erk 1/2 inhibitor U0126 (20 μM), and JNK inhibitor Sp600125 (10 μM) for 1 h before EF stimulation. The same concentration of drug was present during EF exposure in a CO_2_ incubator.

### Quantification of tube-like structures

Images of the tubular structures in Matrigel were taken using the Olympus CKX41 (Olympus, Tokyo, Japan) light microscope and processed with Image-Pro Plus software. At designated time points, six or more images from each experiment were analyzed and the average tube length covered by the cells was calculated.

### Immunofluorescence

Tube-like structures in Matrigel were fixed in 4% paraformaldehyde for 1 h at 37°C followed by permeabilization with 0.1% Triton-X100 for 45 min at room temperature and blocked with 3% BSA for 1.5 h at 37°C. The fixed structures were incubated with rabbit anti-VEGF (dilution 1:300), anti-VEGFR2 (phospho Y1175) (concentration of 5 μg/ml), anti-Akt (dilution 1: 200), anti-Erk 1/2 (dilution 1: 200), anti-p38 MAPK (dilution 1:400), or anti-JNK (dilution 1:200) antibodies, directed against the active (phosphorylated) form of the proteins (Cell Signaling Technology or Abcam) for 1.5 h at 37°C followed by a similar incubation with a secondary goat anti-rabbit DyeLight™ 680-labeled antibody (1:1000, KPL). The cells were stained with DAPI (Invitrogen) and mounted with Vectashield (Vector Laboratories, Burlingame, USA). Images were taken with the Olympus confocal microscope FV1000.

### Statistical analysis

The data were analyzed with SPSS16.0 (SPSS Inc., Chicago, USA). For morphometric analysis, tube-like structures were measured in each of six or more images in duplicate from at least three separate experiments. For protein expression or activation assessments, three separate experiments were performed. Means were compared using one-way analysis of variance (ANOVA) in group comparison. Two-tailed Student's *t*-test for unpaired data was applied as appropriate. A value of *P*<0.05 was considered statistically significant.

## Supplementary Material

Supplementary information
