# Supplementary material for: Physiological electric field works via the VEGF receptor to stimulate neovessel formation of vascular endothelial cells in a 3D environment
Source: Biol Open. 2018 Sep 15;7(9):bio035204. doi: 10.1242/bio.035204 (PMC6176943; doi:10.1242/bio.035204)
Supplement: Supplementary information [file biolopen-7-035204-s1.pdf]

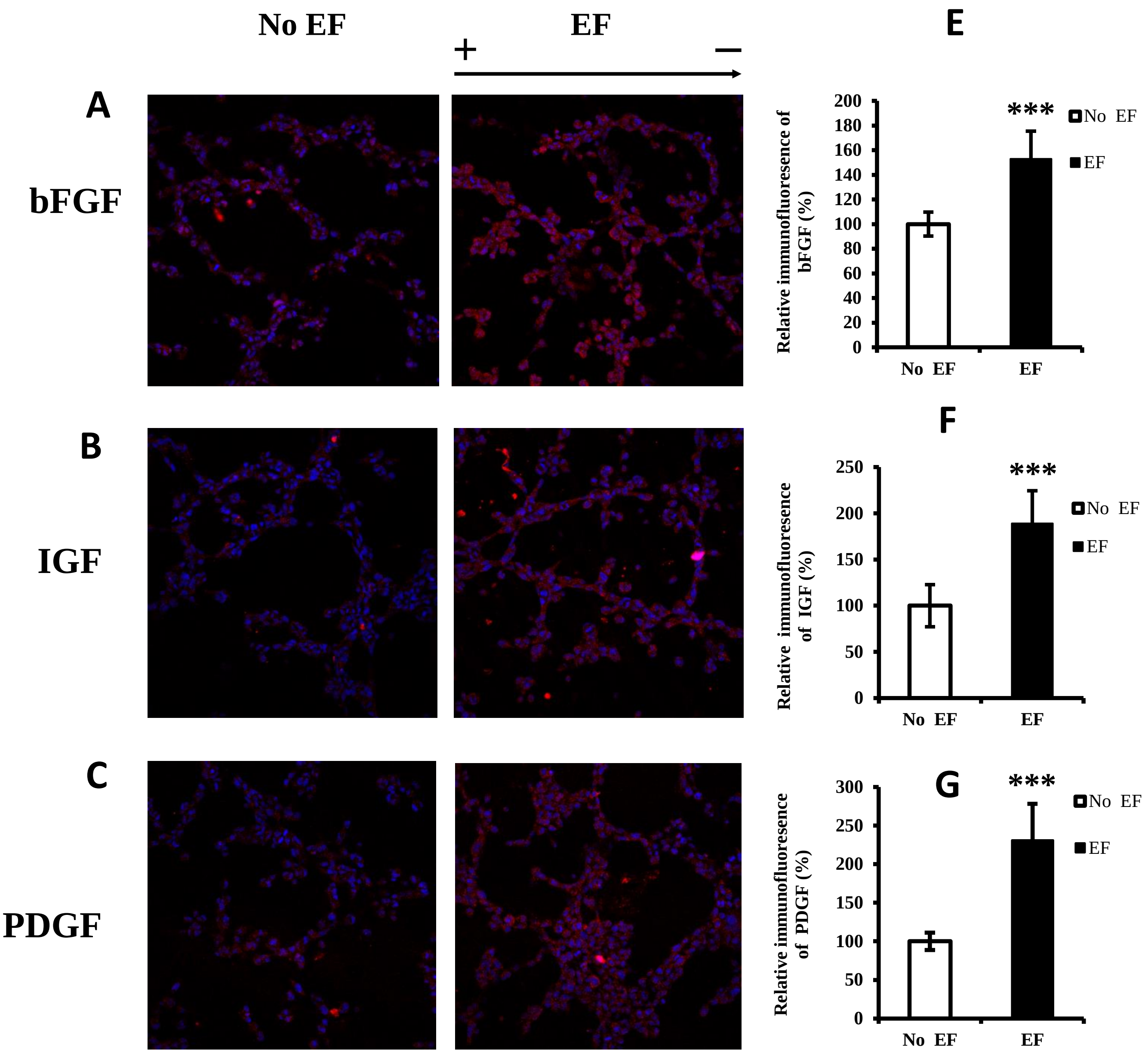

Figure S1. Increase of bFGF, IGF and PDGF expression upon EF treatment of HUVECs cultured in 3D. HUVECs were treated with an electrical field (150 mV/mm). After 6 h, they were fixed and stained with bFGF, IGF and PDGF antibodies. Protein expressions were quantified by confocal laser scanning microscopy. The images show representative immunolabeled tube-like structures. The histogram depicts the relative immunofluorescence of the bFGF, IGF and PDGF proteins. The error bars represent the S.E. ***P<0.001, significantly different from the untreated control. Initial magnification of the images: 200X.

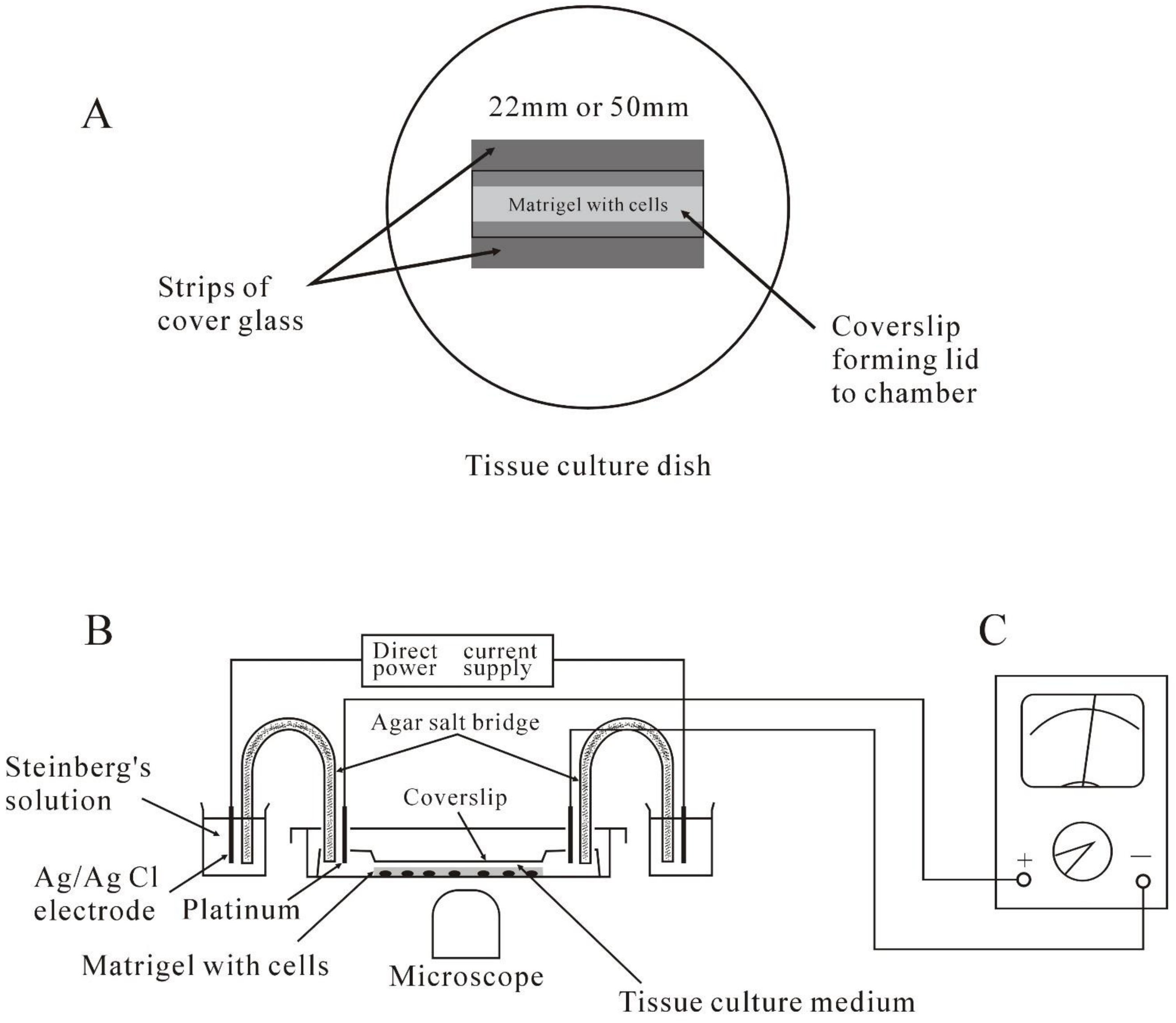

Figure S2. Schematic diagram showing the experimental design of the culture chamber and field application. (A) Top-down view of a chamber constructed within a plastic tissue culture dish. (B) side-on view including the DC power supply and Ag /AgCl electrodes isolated from the culture chamber using agar-gelled salt bridges. (C) Measurement of an EF using an electric meter.

Biology Open • Supplementary information

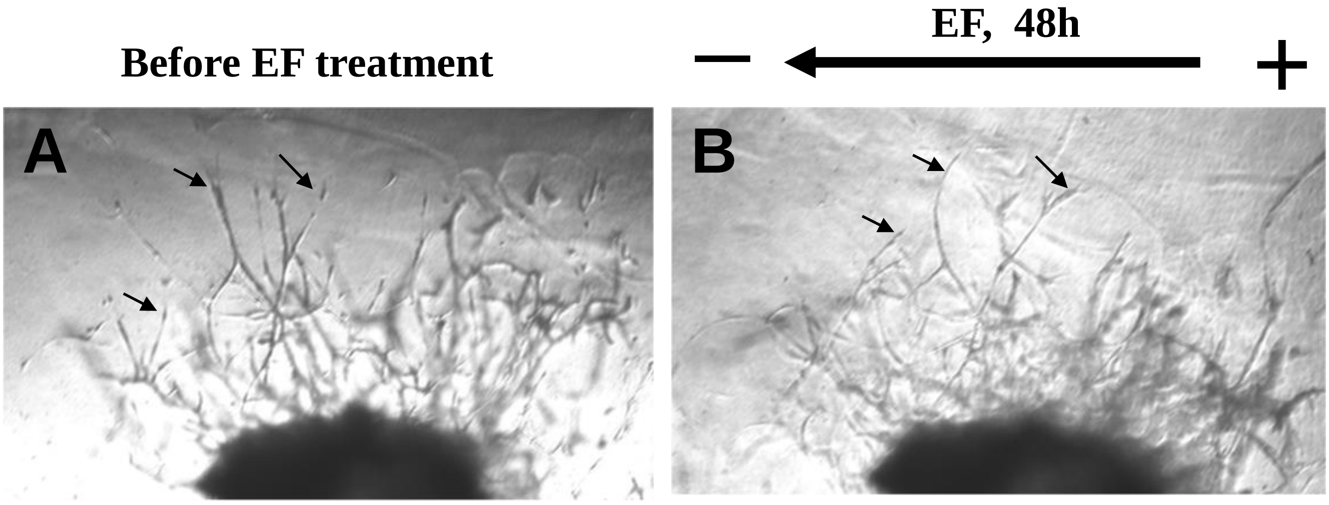

Figure S3. A mouse aortic ring embedded in Matrigel exposed to an EF of 200 mV/mm (over 48 h) sprouted vessel-like structures that grew toward the anode.

Biology Open • Supplementary information
